# The role of background diet on the effects of eicosapentaenoic acid and docosahexaenoic acid supplementation in healthy pre-menopausal women: a randomized, cross-over, controlled study

**DOI:** 10.1186/s12944-016-0341-1

**Published:** 2016-09-29

**Authors:** Megan Arnold Gomes, Xiaoyuan Jia, Iris Kolenski, Alison M. Duncan, Kelly A. Meckling

**Affiliations:** Department of Human Health and Nutritional Sciences, University of Guelph, Guelph, ON N1G 2W1 Canada

**Keywords:** Omega-3 fatty acids, Estrogen, Estrogen metabolites, Nipple aspirate, Urinary biomarkers

## Abstract

**Background:**

The links between dietary fat intake, polyunsaturated fatty acid intake and breast cancer risk remain equivocal, with some studies pointing to improvements in risk upon omega-3 supplementation. However, the background diet is poorly controlled in most studies, potentially confounding this link. Therefore, this study examined the hypothesis that in order to see the benefits of omega-3 fatty acid supplementation, the background diet must be low in fat.

**Methods:**

Of the 56 healthy, pre-menopausal women randomized to one of two experimental arms, consisting of a two-treatment, randomized, cross-over design, 41 completed the 10 month intervention. The two diet phases (habitual and low-fat) were separated by a washout phase, each lasting 3 menstrual cycles. During each diet phase, women were supplemented with 1.2 g eicosapentaenoic acid + docosahexaenoic acid per day.

**Results:**

Red blood cell fatty acid composition indicated that more eicosapentaenoic acid and docosahexaenoic acid was incorporated in the low-fat diet than the habitual diet, though both diet phases resulted in significant increases in the omega-3 to omega-6 ratio. In the context of omega-3 supplementation in breast cancer risk reduction, we also measured fatty acid incorporation into nipple aspirate fluid. Similar changes to red blood cells were noted in nipple aspirate fluid, with higher incorporation of eicosapentaenoic acid in the low-fat diet phase.

**Conclusions:**

These data suggest that the total level of dietary fat has some direct impact on fatty acid partitioning in addition to the recognized importance of fatty acid ratios, and supports the hypothesis that dietary fat intake must be considered a confounder in supplementation trials. Additionally, we demonstrate that n3 supplementation both reaches and imparts improvements in lipid content and n3:n6 at the target breast tissue.

**Trial registration:**

Trial was been retrospectively registered at clinicaltrials.gov (RegNCT02816125).

## Background

Dietary factors, specifically dietary fat, have been hypothesized to account for the large variation in global breast cancer incidence and the increases amongst migrant populations [1, 2]. Support for an influence of dietary fat on breast cancer rates has been demonstrated in animal experiments [3, 4] and international correlation studies [5, 6], however more recent data from cohort and case–control studies have been equivocal [7, 8]. Therefore, the potential link between dietary fat and risk for breast cancer has been controversial for many years, and continued research in this area has only raised more unanswered questions. Recognizing the risk factors for breast cancer permits the identification of women with increased risk of developing the disease and the potential for intervention to modify the risk both individually or through population based approaches.

Specifically, experimental data has linked breast cancer risk to a high dietary intake of n-6 polyunsaturated fatty acids (PUFAs), particularly when associated with a low intake of n-3 PUFAs [9]. Experimental animal models support this with evidence that n-6 PUFAs enhance breast tumourigenesis and metastasis, while in contrast, n-3 PUFAs inhibit growth of initiated breast cancer cells [9]. In addition to total PUFA amounts, dietary n-6 to n-3 FA ratio (n-6:n-3; total omega-6 fatty acids in the diet to total omega-3 fatty acids in the diet) has been associated with breast cancer risk [10–13] and breast cancer risk has increased in Japanese women over the past four decades correlating with a decrease in the dietary n-3:n-6 PUFAs [14].

Despite compelling evidence relating dietary fat and breast cancer from animal models, mechanistic experiments *in vitro* and ecologic studies, these results are not well supported by available epidemiologic data in humans [15]. A pooled analysis of several cohort studies did not find an association between dietary fat and breast cancer [16]. Recently, the results of the Women’s Health Initiative dietary modification trial demonstrate a weak (non-significant) inverse association between a low-fat diet and the risk of breast cancer [17]. These conflicting results have led to uncertainty over the association of dietary fat and breast cancer and thus in nutritional recommendation for breast cancer prevention [18]. Several confounding variables may be responsible for these conflicting results including: methodological issues with regards to study design, measurement error, improper statistical analysis, dietary assessment tools, and a lack of heterogeneity of dietary fat intake of the study participants [7].

More recent epidemiologic studies have attempted to address some of the methodological limitations that affected earlier studies through the use of validated questionnaires, adjusting estimates for a wider range of potential confounders and examining specific fatty acids and their interrelationships [15]. Although the link between a low-fat diet and breast cancer prevention remains controversial [19–21], the evidence is substantial enough to support prospective studies and clinical trials with the hypothesis that reduced intake of dietary fat will decrease breast cancer risk [19, 22]. The current study attempted to determine whether the n-3 incorporation and n-3:n-6 was influenced by the level of total dietary fat intake in a female population with a family history of breast cancer. We hypothesized that the greatest improvements in lipid profiles from n-3 supplementation would occur in the condition of a low-fat diet background.

## Methods

### Participant recruitment and screening

This intervention study was approved by the Research Ethics Board of the University of Guelph (REB235). Potential participants were recruited between February and August 2004 through newspaper advertisements, pamphlets and posters in doctors’ offices in Guelph, ON and the surrounding community.

Potential participants were screened through a phone or email questionnaire after which they were then given a detailed oral and written outline of the study, answers to frequently asked questions, and a 7-day screening food record. Potential participants were deemed eligible if they were healthy, premenopausal, eumenorrheic women between 20 and 54 years of age who were sedentary or recreationally active, had a body mass index (BMI) of 20–30 kg/m^2^ and a dietary fat intake of 30–40 %. Exclusionary criteria included use of oral contraceptives or hormone therapies; smoking; alcohol consumption greater than 7 drinks per week; highly trained athletes; pregnancy and/or lactation within the previous 6 months; consumption of fish oil capsules within the previous 3 months; and the use of thyroid, hypertensive, oral hypoglycemic or insulin therapy.

Once eligibility was established, participants gave informed written consent and were oriented to the study through an individual meeting with a study coordinator. They were provided with a study handbook that included detailed instructions outlining all study visits, a study calendar according to menstrual cycle days, information about the study supplements, materials to assist with the low-fat diet phase of the study, instructions on how to complete accurate food records, 7-day food record forms, a study diary with instructions, and detailed information about all study sample collections.

### Experimental design

**Fig. 1:**
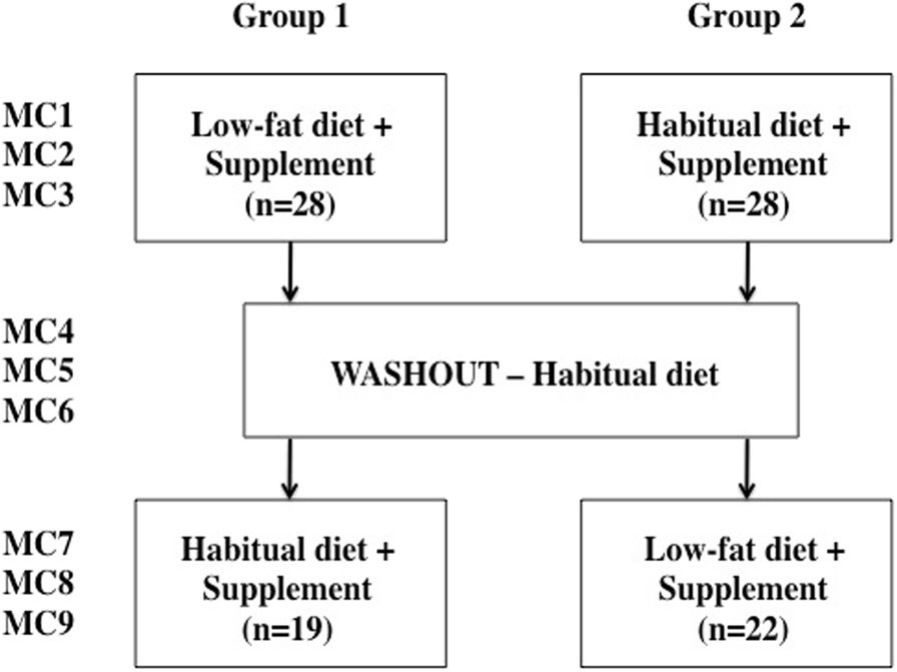
Experimental design and participant flowchart. MC = menstrual cycle

Of the 212 women originally screened, 56 met the eligibility criteria and agreed to participate. These participants were randomized to one of two experimental arms, consisting of a two-treatment, randomized, cross-over design (see Fig. 1). During the eligibility screening, potential participants completed a questionnaire outlining their basic demographic characteristics, pregnancy and breast-feeding history as well as family history of breast cancer and other diseases. Subjects were given an exit questionnaire upon the completion of the study. The purpose of this questionnaire was to assess the feasibility of the low-fat diet, and gain valuable information about the nipple aspirate fluid extraction procedure, subjects menstrual cycle patterns and the study supplement. It also provided subjects with a means to express any concerns or improvements they had with regard to any aspect of the study.

### Study diets and food records

There were two diet phases in the study separated by a washout phase. Subjects were randomized to consume either a low-fat diet or their habitual diet in Phase I. After 3 menstrual cycles (MC) on the Phase I diet, the subjects were asked to consume their habitual diet for 3 menstrual cycles, during which time no supplements were consumed. The participants then crossed over to the other experimental diet, Phase II (habitual or low-fat) for an additional 3 menstrual cycles during which supplement was again consumed.

During the low-fat diet phase, the target fat intake was 20 % of energy or less. The exact quantity of total fat was calculated individually for each participant based on their 7-day habitual food record provided at study entry. Food records were analyzed using ESHA Food Processor (ESHA Research Version 7.11) to calculate energy, total fat, saturated fat, monounsaturated fat, polyunsaturated fat, carbohydrate, protein, dietary fiber, vitamin and mineral intake. A daily fat intake record was provided in the study handbook and filled out each day by participants throughout the low-fat study period. This served as a tool to aid with compliance to the low-fat diet protocol and was reviewed at the weekly nutrition counseling sessions.

Participants completed six 7-day food records throughout the course of the study, including one pre-study screen, three low-fat diet and two habitual diet food records. The food records were completed at different time periods throughout the menstrual cycle to account for possible alterations in food intake patterns. One of the study coordinators contacted each participant individually to provide reminders of when to commence the necessary food records outlined in the study protocol. Food records were analyzed immediately upon receipt by one of the two study coordinators, one of whom was a registered dietician or by one of two undergraduate research students that were trained on the use of Food Processor.

### Study supplements

During both the Phase I (MC1, MC2, MC3) and Phase II (MC7, MC8, MC9) diet intervention periods, all participants consumed 4–500 mg fish oil capsules per day (SEE YOURSELF WELL™ OMEGA-3 Dietary Supplement: See Yourself Well Inc., Leamington, ON) containing 200 mg of eicosapenaenoic acid (EPA) and 100 mg of docosahexaenoic acid (DHA) for a total of 1.2 g n-3/day. On the first day of the fourth menstrual cycle, supplementation was stopped and the participants resumed their habitual diet for a three menstrual cycle washout period (MC4, MC5, MC6). From the first day of their seventh menstrual cycle (MC7), participants restarted the n-3 FA supplementation for another 3 menstrual cycles. Possible side effects of the supplement or diet intervention were discussed at each nutritional counseling session. Supplement compliance was assessed by counting unused capsules and confirmed by analyzing red blood cell phospholipid fatty acid composition (see below).

### Anthropometric measurements

At baseline, height was measured using a metric tape measure (to nearest 0.5 cm) and body weight using a digital scale (to the nearest 0.1 kg; ACCULAB® SV-100), followed by calculation of BMI. During the study, measurements were completed for body weight, body composition, blood pressure, waist and hip circumference according to the schedule summarized in Table 1. Blood pressure was taken on the left arm using an automated cuff digital blood pressure monitor (LifeSource™ UA-767) while the participant remained relaxed and seated. Waist and hip circumference were measured using a flexible tape measure (to the nearest 0.5 cm). The waist circumference measurement was taken at the point of most noticeable narrowing or an indeterminate waist was approximated by taking the girth at the estimated lateral level of the twelfth or lower floating rib and the hip circumference measurement was taken from the widest area of the hip. Body composition was measured using bioelectric impedance analysis (BIA; BodyStat 1500™) in hydrated, fasting participants, as described previously [23].

**Table 1:** Data collection schedule of measurements

	Treatment phase I			Washout phase			Treatment phase II		
	Menstrual cycle			Menstrual cycle			Menstrual cycle		
	1	2	3	4	5	6	7	8	9
Blood Sample	+	+	+	+		+	+	+	+
Body Weight	+	+	+	+		+	+	+	+
Blood Pressure	+	+	+	+		+	+	+	+
Waist: Hip	+	+	+	+		+	+	+	+
Body Composition	+			+		+			+
Urine Collection	+			+		+			+
Nipple Aspirate	+			+		+			+

+ Indicates measurement taken on day 4, 5 or 6 of corresponding menstrual cycle

### Blood collection and analysis

Blood was drawn by venipuncture, after a 12 h fast on day 4, 5 or 6 of the menstrual cycle (for whole blood, red blood cell, serum and plasma separation). In order to minimize diurnal variation of reproductive hormones, the time of blood draw was kept as consistent as possible within each participant. Whole blood and isolated fractions were separated, aliquoted into multiple tubes and stored frozen at −80 °C until analysis.

Serum samples were analyzed in triplicate for estradiol (E2) levels using a competitive enzyme immunoassay (EIA; Cayman Chemical, Ann Arbor, MI) according to manufacturer’s instructions. Three separate analyses were run over the course of the 1.5 years of the trial period. At each point a lab-specific and assay kit control sample was included to reduce inter-assay variability.

Red blood cells were obtained by removing white cells and plasma/serum by centrifugation and stored at −80 °C until analysis. Total red blood cell fatty acid composition was analyzed by a commercial laboratory, Lipid Analytical Labs (Guelph, ON), using a combination of lipid extraction and Gas Chromatographic techniques. Total lipid was extracted according to the methods of Bligh and Dyer [24]. The phospholipid and triglyceride fractions were separated from other lipids by thin–layer chromatography on silica Gel F Redi/Plates (Fisher, Unionville, ON) in a solvent of heptane:isopropyl ether:acetic acid (60:40:3). The origin, containing phospholipids, and the triglyceride fraction were scraped after visualization with 0.1 % aminonaphtholsulfonic acid, and fatty acids were methylated after addition of the fatty acid 17:0 (3 μg) as an internal standard. Methylated fatty acids were analyzed on a Varian 3400 gas–liquid chromatograph (Palo Alto, CA) with a 60-m DB-23 capillary column (0.32 mm internal diameter), as previously described [25].

### Urine collection and analysis

Participants collected the first void of the morning urine samples on day 4, 5 or 6 (the morning of their study visit) of menstrual cycles 1, 4, 6, and 9, and the beginning and end of each treatment period. Participants were provided with 3-l urine collection containers (VWR International, Mississauga, ON) and a urine collection hat (Norfolk Medical Supply, Guelph, ON) to place directly over the toilet seat during urine collection. All urine collection containers included a label, which outlined the urine collection protocol and provided space to record the participant number, date and time of collection. The urine collection protocol required participants to collect all urine produced during the first morning void immediately after waking up, and if urination occurred frequently throughout the night to collect all voids after falling asleep and the subsequent first void of the morning.

Participants were instructed to urinate directly into the urine collection hat and then transfer all urine into the collection container and store it in the refrigerator until reporting for their study visit. Participants collected urine in a fasted state and were instructed not to consume alcohol for 24 h prior to urine collection. The time of urine collection was kept consistent within each participant to minimize diurnal variation of reproductive hormones. Upon delivery, the total volume of urine and collection date and time were recorded. The urine was mixed with ascorbic acid (1 mg/mL of urine; Fisher Scientific) to prevent oxidation of labile compounds, and then aliquoted into four separate 15 mL conical tip tubes (Starstedt, Montreal, QC) and frozen at −80 °C for future analysis.

Urine samples were thawed and analyzed in triplicate for 2-hydroxyestrone (2-OHE1) and 16-α-hydroxyestrone (16-α-OHE1) using a competitive solid-phase enzyme immunoassay (EIA, Immuna Care Corp, Bethlehem, PA). Lab-specific and assay kit control samples were included in each kit in order to reduce inter-assay variability.

### Nipple aspirate fluid collection and analysis

Nipple aspirate fluid (NAF) was collected on day 4, 5 or 6 (the morning of their study visit) of menstrual cycles 1, 4, 6, and 9, and at the beginning and end of each treatment period, using a FirstCyte™ Aspirator (FirstCyte™ Aspirator, Cytyc Health Corporation). The aspirator consisted of a clear, rigid polycarbonate cup with foam liner, attached to a 20 mL syringe which was used to pull a gentle suction to express NAF using a modification of techniques that have been described previously [26, 27] and through direct communication via the telephone with a researcher currently performing the procedure. Nipple aspiration took place in a private room with participants relaxed and seated in a comfortable upright position after cleaning, warming and gently massaging the breast. The cup attached to the syringe was placed directly over the centre of the nipple and suction was applied by withdrawing the plunger of the syringe to the 5–15 mL range, as tolerated, and held for 20 s or until the participant experienced discomfort. If NAF did not appear, the suction was repeated up to five times until fluid was obtained. When NAF appeared, the aspirator was carefully removed and the fluid droplets were collected in capillary tubes. The entire procedure was repeated on the opposite breast. The NAF, contained within capillary tubes, was stored in microcentrifuge tubes and frozen at −80 °C for future analysis. When NAF did not appear after five repeated attempts, the procedure was repeated during the following (MC4) study visit. If NAF was not produced after two separate attempts, the participant was designated a non-secretor and the procedure was discontinued. Of the women who participated, only 45 % were able to provide sufficient NAF for analysis. Of the entire cohort, 15 gave sufficient material for fatty acid analysis at baseline, during the low-fat supplement or the habitual supplement phases (analyzed in the same manner as RBCs described above).

Nipple aspirate samples were analyzed in triplicate for estradiol levels using a competitive enzyme immunoassay (EIA; Cayman Chemical, Ann Arbor, MI) according to manufacturer’s instructions. In some cases samples were diluted in buffered saline to achieve concentrations within the standard curve. Because of the different volumes of NAF collected from each participant, estradiol values were normalized to the protein concentration of the NAF determined the method of Bradford [28]. Because the NAF samples were all analyzed at the end of the trial, serum samples (from the same participants, at the same time point in the trial) were re-run at the same time to control for changes in hormone levels with storage time. At each point a lab-specific and assay kit control sample was included to reduce inter-assay variability.

### Statistical analysis

The anthropometric measurements and food records were compared between baseline and after supplementation administration on both the low-fat and habitual diets. Serum estradiol and urinary estrogen metabolic ratios were analyzed using Analysis of Variance where treatment and order were each predictor variables because of the cross-over design of the trial. These analyses indicated that there was no significant effect of order and therefore this block was removed for all subsequent analysis. Estradiol, urinary estrogen metabolites and NAF fatty acid composition differences were analyzed by one-way ANOVA with repeated measures using SPSS version 12 for Windows. FA profiles of red blood cells in the low-fat diet with supplementation were compared to baseline values by using a paired t-test (*n* = 41) and differences between the two supplement phases by paired t-test (*n* = 8). Differences were considered significant when *p* < 0.05.

## Results

Of the 56 participants randomized to one of the two study arms, 47 actually began the study protocol with the remaining 9 giving no reason for dropping out. During the subsequent 9–10 months, four participants were removed from the study because of non-compliance with the dietary protocol, one left for unknown reasons and one withdrew because of pregnancy. Characteristics of the 41 participants completed the intervention are summarized in Table 2. The average age of the participants was 37.7 ± 1.4 years. Over 94 % of the participants were Caucasian in ethnic origin and average BMI was 23.4 ± 0.4 kg/m^2^. To assess compliance to the study protocol, 7-day diet records were periodically collected and analyzed and capsules not consumed were recorded at each study visit. Excellent compliance in consuming the n-3 supplement during the two diet phases was observed (~92 %; Table 3). The average menstrual cycle length was 28.5 days (SD = 3.1) and was not different between the habitual and low-fat diets (*p* = 0.82). Menstrual cycle length ranged from 24 to 42 days meaning that the supplementation period for each diet phase could have been as few as 72 days and as many as 126 days. This is a very large potential difference in the supplementation time but because of the small sample size, no effect of cycle length was found when examining the changes in fatty acid composition (see below).

**Table 2:** Baseline characteristics of participants who completed both phases of the study protocol

	Mean ± SE	Range
Age (years)	37.7 ± 1.4	20–54
Body weight (kg)	64.1 ± 1.5	46.6–83.7
Body Mass Index, BMI (kg/m^2^)	23.4 ± 0.4	18.9–29.9
Age at menarche (years)	13.0 ± 0.4	11–16
Ethnicity		
White (%)	93	
Non-white (%)	7	
Full term pregnancy at least once		
Yes (%)	61	
No (%)	39	
Breast-fed infant		
Yes (%)	96	
No (%)	4	
Age at first live birth (years)	26.9 ± 4.6	19–38
Family history of breast cancer		
Yes (%)	63.3	
No (%)	36.7	
Family history of hypertension		
Yes (%)	56.1	
No (%)	43.9

**Table 3:** Compliance to study omega-3 fatty acid supplements

	Missed capsules/MC	Missed days/MC	Compliance^a^	*p*-value
	(*n* ± SE)	(*n* ± SE)		
Low-fat diet phase	8.3 ± 3.5	2.1 ± 0.9	92.5 %	0.88
Habitual diet phase	8.9 ± 2.0	2.3 ± 0.5	91.8 %	

^a^compliance was calculated as [(# capsules dispensed − # capsules returned)/capsules dispensed] × 100 %

The anthropometric measurements of 41 participants taken at both the beginning and end of each intervention period are shown in Table 4. After the low-fat diet phase, women had a significant reduction in: body weight, BMI, waist circumference, hip circumference, waist to hip circumference ratio and percentage body fat. Following the habitual diet phase, weight and BMI both significantly increased but there were no differences in other anthropometric measures.

**Table 4:** Measures of participants before and after the low-fat and habitual diet phases

	Low-fat diet phase			Habitual diet phase		
	(Mean ± SE; *n* = 41)			(Mean ± SE; *n* = 41)		
	Before	After	p1	Before	After	p2
Body Weight (kg)	63.5 ± 1.4	61.7 ± 1.3	<0.001	62.2 ± 1.3	63.3 ± 1.3	<0.001
BMI (kg/m^2^)	23.4 ± 0.5	22.7 ± 0.4	<0.001	22.9 ± 0.4	23.4 ± 0.4	<0.001
Waist (cm)	76.2 ± 1.1	74.6 ± 1.0	<0.001	75.8 ± 1.0	76.3 ± 1.0	0.93
Hip (cm)	101.0 ± 1.0	99.4 ± 0.9	<0.001	100.2 ± 1.1	101.3 ± 0.9	0.44
Waist:Hip	0.76 ± 0.05	0.75 ± 0.04	<0.05	0.75 ± 0.004	0.75 ± 0.04	1.0
Body fat (%)	30.2 ± 0.8	28.6 ± 0.8	<0.001	29.8 ± 0.9	30.3 ± 0.8	0.68
Lean mass (%)	18.9 ± 0.5	19.0 ± 0.5	NS	19.3 ± 0.5	19.3 ± 0.4	1.0

p1, significance using a paired t-test before vs. after low-fat diet. p2, significance of the paired t-test before vs. after habitual diet

There was no significant difference in total energy intake between the low-fat and habitual diet phases (Table 5), despite the fact that a significant loss of body weight was recorded during the low-fat phase. As expected, fat intake was lower and carbohydrate intake higher in the low-fat versus the baseline or habitual diet phases. On average, habitual fat intake was 35 % of calories and decreased to 22 % during the low-fat phase. There were no significant differences in protein intake between the low-fat and habitual phases suggesting that the decreased fat intake was largely compensated for by an increase in carbohydrate consumption. While there were no significant differences in vitamins A or D intake between the different diet phases, vitamin E intake was significantly lower in the low-fat diet phase. Calcium intake was also similar between the diets. Before taking into account supplementation, both n-3 and n-6 fatty acid intake were significantly lower in the low-fat diet compared to habitual diet, however, no difference was found in the ratio of n-3 to n-6 fatty acids between these two phases (Table 5). With 1200 mg of EPA plus DHA in the supplements, the n-3 fatty acid (FA) intake from all sources was significantly higher in both intervention phases compared to the washout phase (1.86 g/d in low-fat diet and 2.26 g/d in habitual diet vs. 1.06 g/d during washout), and was accompanied by a significant increase in the n-3:n-6 (Table 5).

**Table 5:** Average daily intake of nutrients in the low-fat diet and habitual diet phases (*n* = 38^a^)

	Habitual diet	Low-fat diet	*p*-value
	(Mean ± SE; *n* = 38^a^)	(Mean ± SE; *n* = 38)	
Energy (kcal) (diet alone)^b^	1884 ± 42	1761 ± 41	0.17
Energy (kcal) (diet + supplement)^c^	1902 ± 45	1790 ± 26	0.22
Carbohydrate (g)	240 ± 7	269 ± 11	<0.05
Protein (g)	77 ± 3	79 ± 2	0.58
Fat (g) (diet alone)	66 ± 2	39 ± 2	<0.01
Fat (diet + supplement)	68 ± 2	42 ± 2	<0.01
Fat total (% energy)	35.7 ± 0.8	22.4 ± 0.5	<0.01
Vitamin A (IU)	4796 ± 583	4137 ± 585	0.43
Vitamin E (mg)	10.0 ± 0.9	7.4 ± 0.5	<0.05
Vitamin D (IU)	204 ± 21	201 ± 19	0.92
Calcium (mg)	937 ± 61	850 ± 45	0.41
n-3 FAs (g) (diet alone)	1.06 ± 0.08	0.64 ± 0.05	<0.01
n-3 FAs (g) (diet + supplement)	2.26 ± 0.08	1.86 ± 0.07	<0.01
n-6 FAs (g)	7.0 ± 0.5	4.7 ± 0.4	<0.01
n-3:n-6 (diet alone)	0.15 ± 0.02	0.14 ± 0.02	0.37
n-3:n-6 (diet + supplement)	0.32 ± 0.02	0.40 ± 0.03	0.11

*p*-value, paired t-test between habitual diet and low-fat diet; ^a^three participants had incomplete food records; ^b^Diet alone, ^c^Food record analyzed from dietary source without supplement. Diet + supplement, Food record analyzed from diet and DHA-EPA supplement

**Fig. 2:**
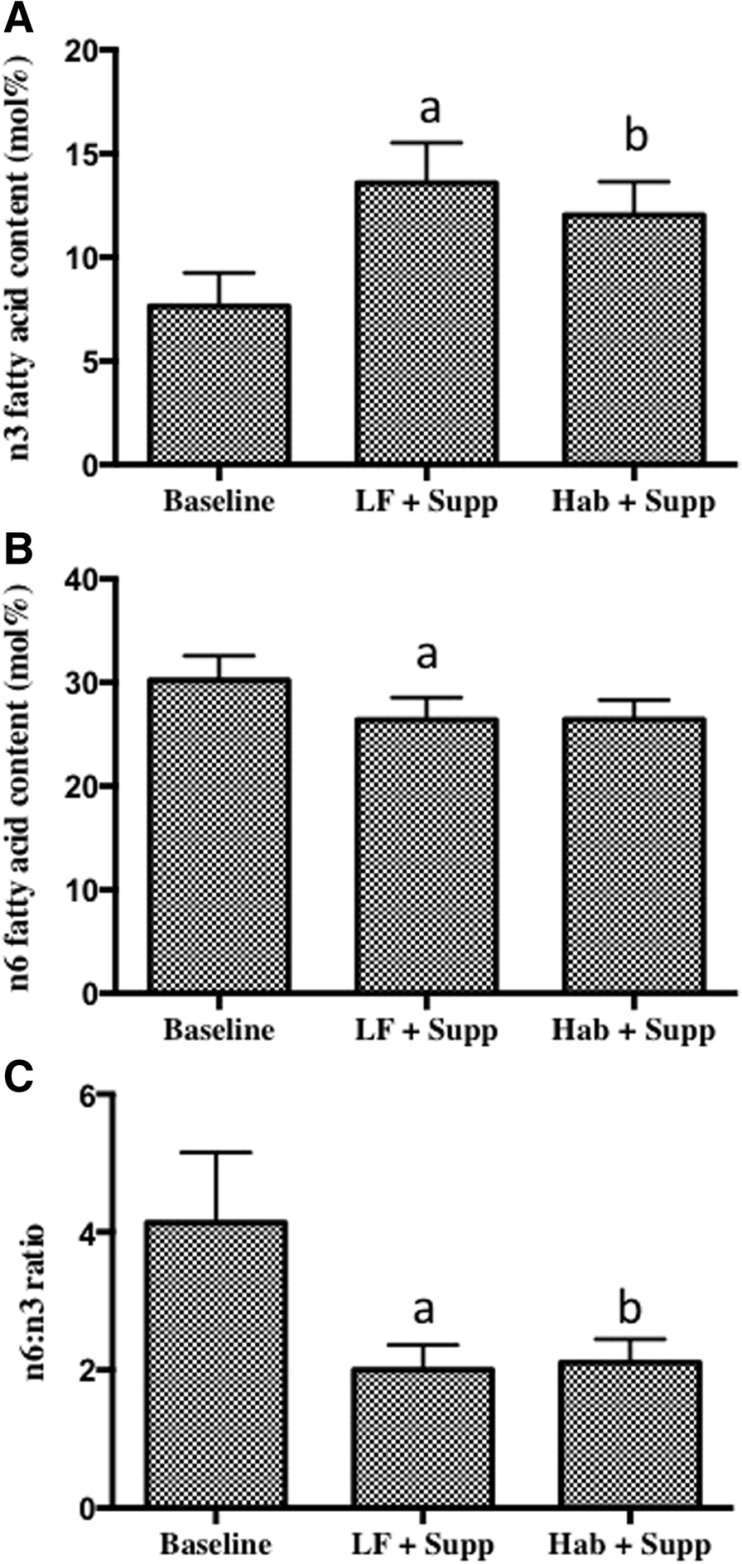
Omega-3 and omega-6 content in red blood cells. **a** Omega-3 (*n* = 41), **b** omega-6 (*n* = 8) and **c** n-3:n6 content in red blood cell membrane phospholipids. Data are expressed as a percentage of total fatty acids ± standard error. *a* = significantly different from baseline, *b* = significantly different from low fat diet phase by paired t-test (*p* < 0.05)

The mean values of the individual FA phospholipid (PL) moieties in red blood cell (RBC) membranes are presented in Table 6. During the low-fat diet phase, n-3 supplementation resulted in a significant increase in both DHA and EPA, and a significant decrease in arachidonic acid (AA). The total unsaturated FA level significantly increased from 38.0 to 40.1 %. Additional significant changes in FA composition of RBC during the low fat diet (LFD) phase included decreased 18:2n-6, 20:2n-6, 20:3n-6, and 22:4n-6 as well as the long chain saturated FA 22:0, long chain monounsaturated fatty acids (MUFAs) 20:1, 22:1, 24:1 and increased shorter chain MUFAs including C14:1, C16:1, C 18:1. While many of the fatty acid changes observed in the low-fat supplementation phase were mirrored in the habitual supplement diet phase, there were some notable differences. In particular, DHA and EPA were significantly lower in the habitual diet phase than that observed in the low-fat intervention period. This resulted in a significantly higher total percentage of n-3 in the LFD and a significantly higher ratio of n-3:n-6. However, compared to the habitual diet without supplementation, both supplementation periods resulted in substantial decreases in total n-6, increases in n-3 and dramatic improvements in the n-3:n-6 ratio (Fig. 2; 0.25 in habitual diet, 0.51 in low-fat supplement and 0.46 in habitual supplement) and the ratio of AA:EPA (19.0 in habitual diet, 4.8 in low-fat supplement and 5.3 in habitual supplement).

**Table 6:** Fatty acid composition (% by weight) in red blood cell membranes

	Baseline (mean ± SE)	Low-fat diet (mean ± SE)	p1	Habitual diet (mean ± SE)	p2
	(*n* = 41)	(*n* = 41)		(*n* = 8)	
C14:0	0.38 ± 0.07	0.36 ± 0.07	0.84	0.40 ± 0.04	0.81
C14:1	0.23 ± 0.18	0.39 ± 0.07	<0.01	0.45 ± 0.14	0.35
C15:0	0.22 ± 0.04	0.20 ± 003	0.69	0.19 ± 0.03	0.56
C16:0	23.59 ± 1.31	23.71 ± 1.44	0.95	22.6 ± 1.31	0.60
C16:1	0.22 ± 0.07	0.30 ± 0.10	<0.01	0.32 ± 0.04	0.24
C18:0	12.15 ± 0.81	12.04 ± 0.85	0.92	14.07 ± 0.51	<0.05
C18:1	17.04 ± 1.07	17.44 ± 1.25	<0.05	18.68 ± 0.43	0.18
C18:2n-6	12.20 ± 1.53	10.56 ± 2.13	<0.01	10.56 ± 0.86	0.36
C18:3n-6	0.04 ± 0.04	0.04 ± 0.03	1.0	0.03 ± 0.02	1.0
C18:3n-3	0.19 ± 0.06	0.18 ± 0.06	0.91	0.22 ± 0.06	0.73
C18:4n-3	0.02 ± 0.02	0.02 ± 0.02	1.0	0.10 ± 0.02	<0.05
C20:0	0.20 ± 0.15	0.16 ± 0.07	0.81	0.05 ± 0.02	<0.05
C20:1	0.31 ± 0.14	0.23 ± 0.07	<0.01	0.23 ± 0.04	0.59
C20:2n-6	0.13 ± 0.12	0.02 ± 0.02	<0.01	0.21 ± 0.06	<0.05
C20:3n-6	1.43 ± 0.34	1.25 ± 0.27	<0.01	1.34 ± 0.23	0.83
C20:4n-6 (AA)	12.96 ± 1.28	11.98 ± 1.55	<0.01	12.16 ± 1.21	0.66
C20:3n-3	0.01 ± 0.02	0.01 ± 0.01	1.0	0.01 ± 0.01	1.0
C20:4n-3	0.05 ± 0.04	0.05 ± 0.03	1.0	0.05 ± 0.05	1.0
C20:5n-3 (EPA)	0.80 ± 0.35	2.64 ± 0.62	<0.01	2.27 ± 0.4	*P* < 0.05
C22:0	1.15 ± 0.31	0.76 ± 0.19	<0.05	0.00 ± 0.00	<0.01
C22:1	0.99 ± 1.00	0.44 ± 0.16	<0.01	0.43 ± 0.33	0.60
C22:2n-6	0.10 ± 0.07	0.10 ± 0.04	1.0	0.00 ± 0.00	<0.01
C22:4n-6	3.08 ± 0.59	2.39 ± 0.53	<0.01	2.11 ± 0.44	0.21
C22:5n-6	0.33 ± 0.26	0.38 ± 0.66	0.94	0.02 ± 0.03	0.26
C22:5n-3	2.25 ± 0.44	3.74 ± 0.72	<0.01	3.70 ± 0.46	0.07
C22:6n-3 (DHA)	4.37 ± 1.09	6.91 ± 1.03	<0.01	6.08 ± 1.04	<0.05
C24:0	1.74 ± 1.30	1.89 ± 1.33	0.94	1.47 ± 0.16	<0.01
C24:1	3.78 ± 2.42	1.93 ± 0.45	<0.01	2.59 ± 0.46	<0.05
Saturated	39.44 ± 2.30	39.12 ± 1.71	0.91	38.75 ± 1.81	0.82
MUFA	22.58 ± 3.13	20.73 ± 1.48	0.59	21.04 ± 1.41	<0.05
PUFA	37.97 ± 2.71	40.13 ± 2.61	<0.01	39.94 ± 2.95	0.63
n-3	7.63 ± 1.61	13.57 ± 1.96	<0.01	12.03 ± 1.62	<0.05
n-6	30.20 ± 2.38	26.40 ± 2.14	<0.01	26.41 ± 1.90	0.23
n-3:n-6	0.25 ± 0.02	0.51 ± 0.02	<0.01	0.46 ± 0.007	<0.05
AA/EPA	19.01 ± 7.77	4.86 ± 1.74	<0.01	5.30 ± 1.04	0.10

Data are expressed as a percentage of total fatty acids ± standard error. p1, significance of paired t-test of baseline vs. after low-fat diet, p2, significance of paired t-test of after low-fat diet vs. after habitual diet

**Fig. 3:**
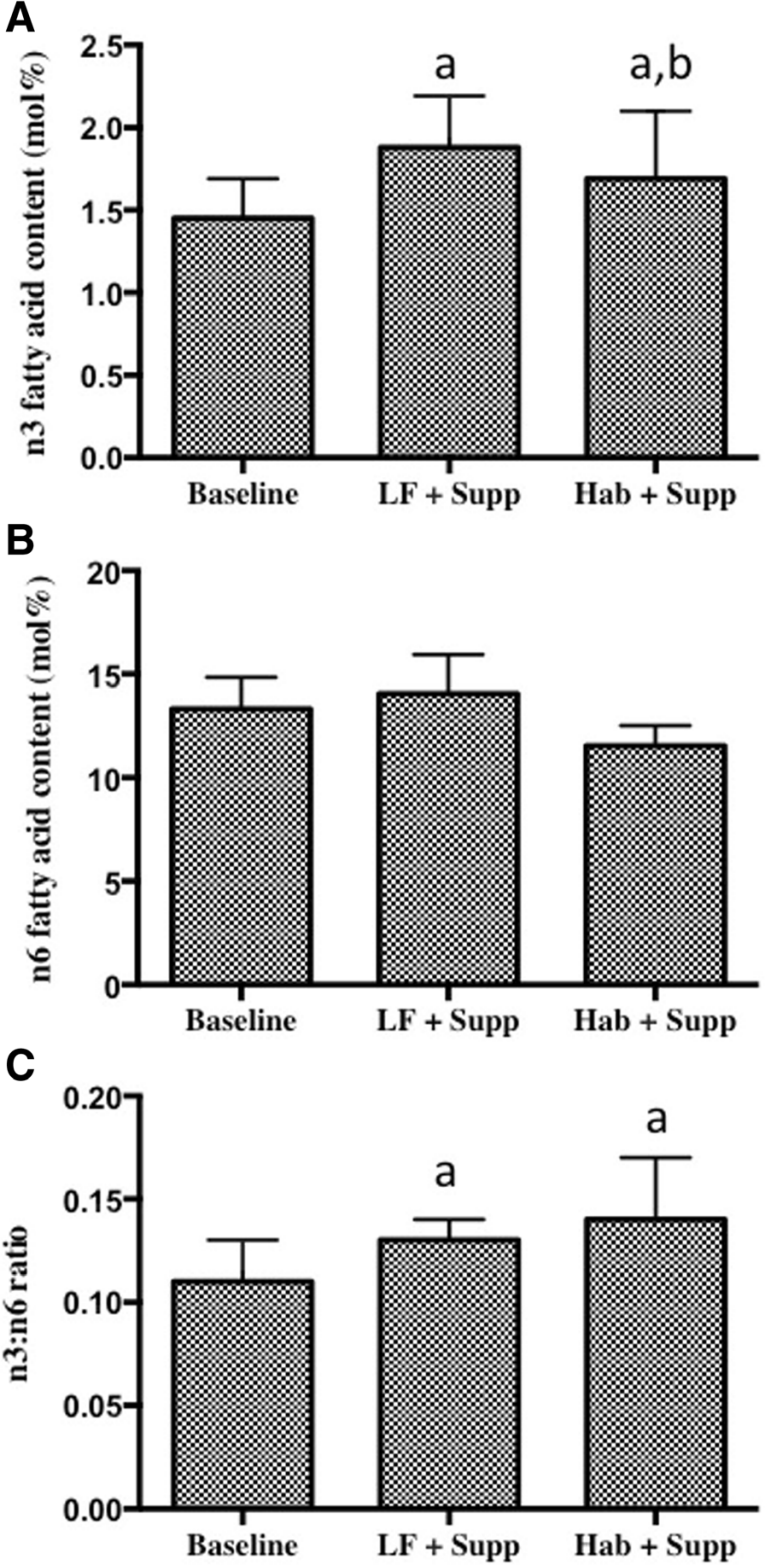
Omega-3 and omega-6 content in nipple aspirate fluid. **a** Omega-3, **b** omega-6 and **c** n-3:n-6 (*n* = 15) content in nipple aspirate fluid. Data are expressed as a percentage of total fatty acids ± standard error. *a* = different from baseline, *b* = different from LFD + supplement by ANOVA and Tukey's post-hoc test (*p* < 0.05)

The fatty acid composition of nipple aspirate total phospholipids is shown in Table 7. There were a number of small, but significant effects of supplementation on NAF composition, demonstrating that supplementation reached the target tissue. In particular, there was a decrease in AA in the habitual supplement phase and in 22:4n-6 in the low-fat supplement diet phase. Similar to the changes in RBC fatty acids, there were significant increases in both EPA and DHA in the supplement phases compared to the habitual phase without supplementation. Again the increase in EPA was significantly larger in the low-fat diet phase compared to the habitual supplement phase. This resulted in a significant increase in n-3:n-6 ratio in both supplement phases and similar decreases in the AA:EPA ratios (Fig. 3). However, there were substantial differences in the major fatty acids of NAF PL compared to RBC PL. For example, C14:0 represented approximately 0.4 % of RBC PL and 9 % in NAF. Total saturated fatty acids were similar between tissues (approximately 40 %), but MUFAs represented 45 % in NAF and only 22 % in RBC PL and PUFAs 15 % in NAF and 40 % in RBC PL.

**Table 7:** Fatty acid composition of NAF phospholipids^1,2^

	Baseline (mean ± SE; *n* = 15)	Low fat diet (mean ± SE; *n* = 15)	Habitual diet (mean ± SE; *n* = 15)
C14:0	10.02 ± 1.37	9.12 ± 0.86	8.83 ± 1.06
C14:1	0.32 ± 0.05	0.31 ± 0.08	0.31 ± 0.06
C15:0	1.65 ± 0.51^a^	1.29 ± 0.35^b^	1.38 ± 0.35^b^
C16:0	18.15 ± 2.81	21.0 ± 1.17	21.98 ± 1.07
C16:1	5.03 ± 0.99^a^	4.20 ± 0.78^b^	4.71 ± 1.20^ab^
C18:0	8.4 ± 0.33	8.29 ± 0.63	9.04 ± 1.01
C18:1	39.1 ± 1.9^a^	37.66 ± 1.46^b^	37.11 ± 1.03^b^
C18:2n-6	11.6 ± 1.4	11.93 ± 1.62	10.12 ± 0.98
C18:3n-6	0.01 ± 0.01	0.03 ± 0.01	0.08 ± 0.03
C18:3n-3	1.03 ± 0.17^a^	0.65 ± 0.14^b^	0.79 ± 0.19^ab^
C18:4n-3	0.008 ± 0.008^a^	0.11 ± 0.10^b^	0.06 ± 0.04^b^
C20:0	0.51 ± 0.10	0.57 ± 0.14	1.14 ± 0.41
C20:1	0.72 ± 0.14	0.82 ± 0.19	1.22 ± 0.27
C20:2n-6	0.29 ± 0.06	0.22 ± 0.06	0.25 ± 0.07
C20:3n-6	0.32 ± 0.07^a^	0.58 ± 0.16^b^	0.47 ± 0.16^b^
C20:4n-6 AA	0.94 ± 0.19^a^	1.21 ± 0.38^ab^	0.52 ± 0.17^b^
C20:3n-3	0.03 ± 0.01	0.05 ± 0.04	0.03 ± 0.01
C20:4n-3	0.04 ± 0.02	0.03 ± 0.01	0.03 ± 0.01
C20:5n-3 EPA	0.06 ± 0.03^a^	0.42 ± 0.20^c^	0.18 ± 0.06^b^
C22:0	0.18 ± 0.04^a^	0.12 ± 0.05^b^	0.21 ± 0.12^a^
C22.1	0.26 ± 0.09	0.22 ± 0.08	0.28 ± 0.10
C22:2n-6	0.003 ± 0.002	0.05 ± 0.03	0.003 ± 0.003
C22:4n-6	0.08 ± 0.03^a^	0.04 ± 0.01^b^	0.09 ± 0.03^a^
C22:5n-3	0.09 ± 0.03^a^	0.16 ± 0.05^b^	0.19 ± 0.05^b^
C22:6n-3 DHA	0.19 ± 0.05^a^	0.47 ± 0.11^b^	0.42 ± 0.14^b^
C24:0	0.50 ± 0.14^a^	0.22 ± 0.08^b^	0.06 ± 0.03^c^
C24:1	0.64 ± 0.18^a^	0.23 ± 0.08^b^	0.51 ± 0.26^ab^
Saturated	39.18 ± 2.74^a^	40.62 ± 1.47^ab^	42.64 ± 2.33^b^
MUFA	46.04 ± 2.61^a^	43.45 ± 1.93^b^	44.13 ± 1.77^b^
PUFA	14.78 ± 1.59	15.93 ± 2.14	13.23 ± 1.23
Total n-3	1.45 ± 0.24^a^	1.88 ± 0.31^c^	1.69 ± 0.41^b^
Total n-6	13.32 ± 1.52	14.05 ± 1.90	11.54 ± 0.98
n-3:n-6	0.11 ± 0.02^a^	0.13 ± 0.01^b^	0.14 ± 0.03^b^
AA/EPA	2.04 ± 0.98^a^	1.50 ± 0.37^b^	1.64 ± 0.59^b^

^1^Fatty acids are reported as mol % of total fatty acids in phospholipids ± Standard Error of the mean

^2^Values in a row not sharing a letter are significantly different (*p* < 0.05) by ANOVA followed by Tukey's post-hoc test

**Fig. 4:**
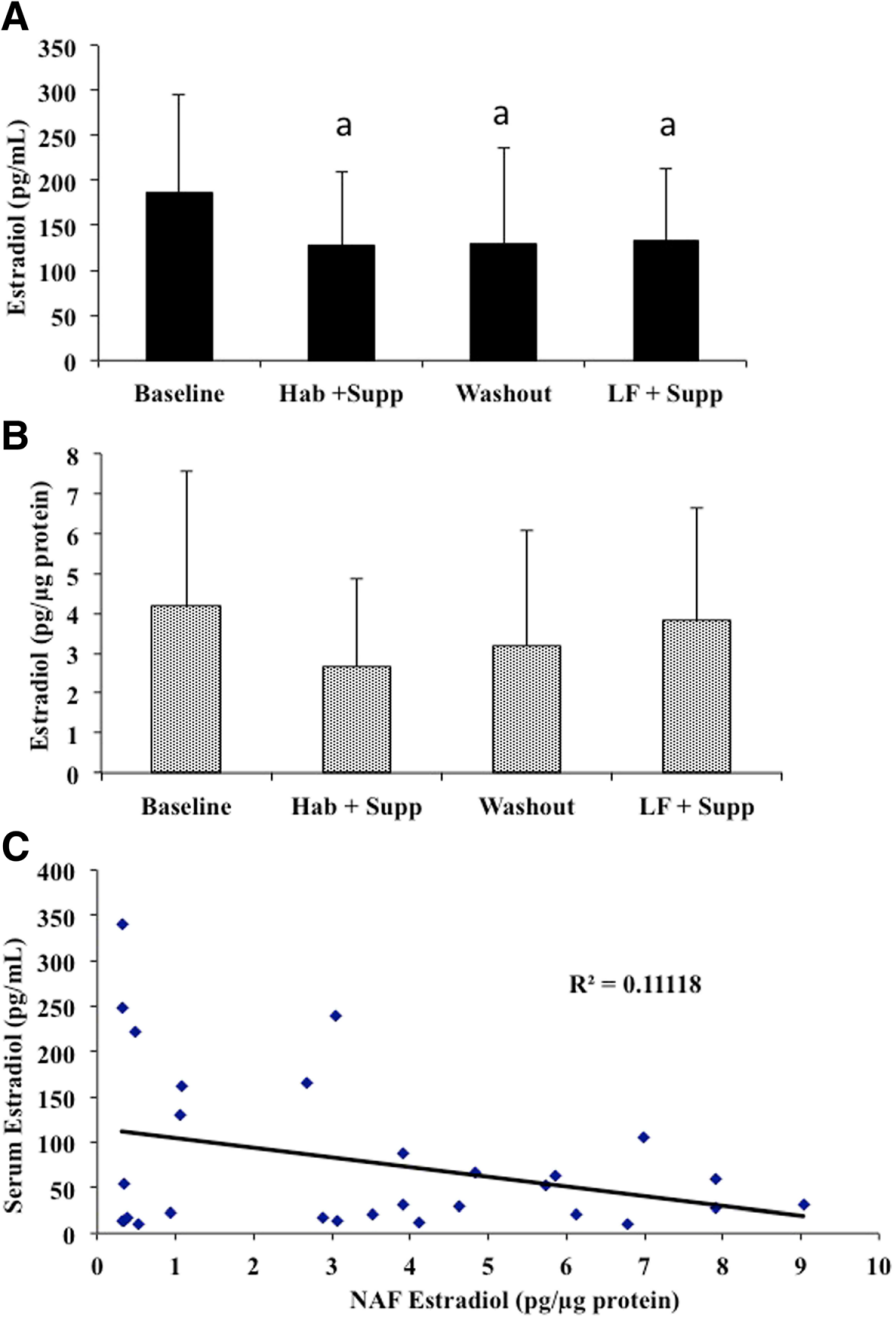
Blood serum and NAF estrogen levels. 17-β-estradiol was measured by radioimmunoassay at baseline and 3 MC after each intervention or washout period. **a** Average E2 levels ± SE in serum (*n* = 41). **b** Average E2 levels ± SE in NAF expressed per μg protein (*n* = 15). **c** correlation between serum and NAF E2 concentrations. Points represent the serum E2 value plotted as a function of the corresponding NAF value. *a* = different from baseline by ANOVA and Tukey's post-hoc test (*p* < 0.05)

**Fig. 5:**
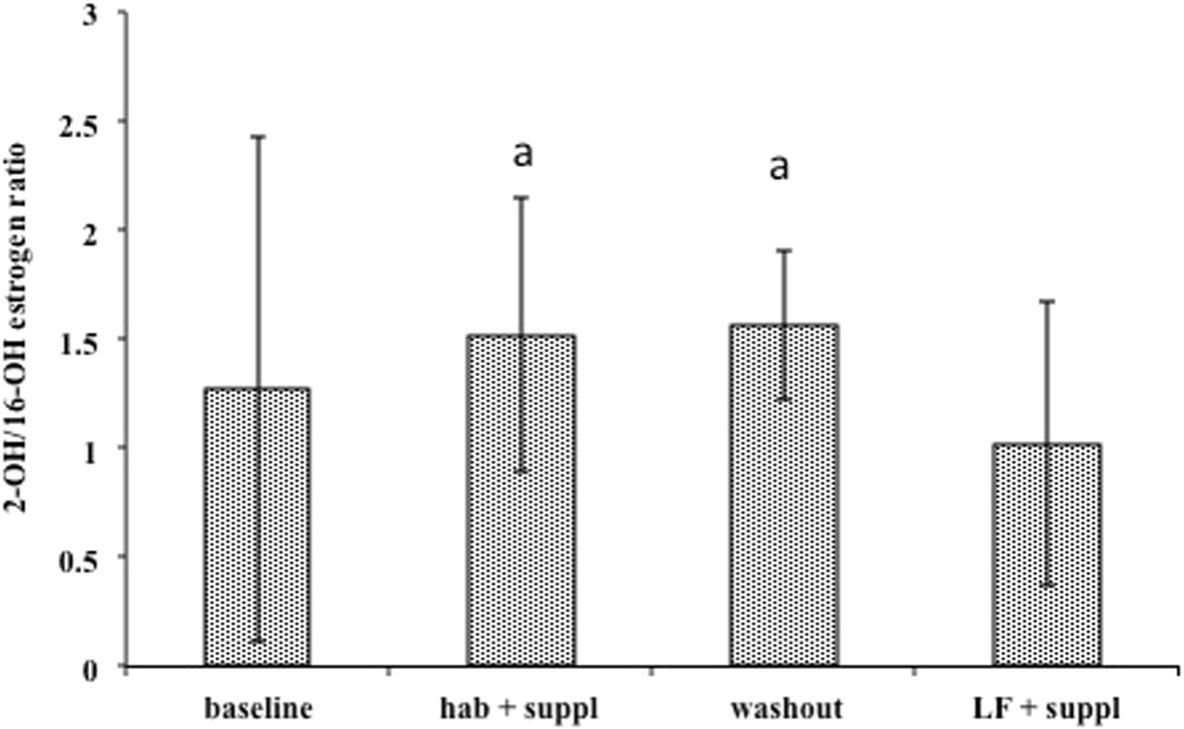
Relative concentrations of urinary estrogen metabolites 2-OHE1 and 16α-EOE1. Complete morning voids were collected on the same day of the menstrual cycle each month. Portions were frozen at −80 °C until analysis. Freshly thawed samples (*n* = 34) were thawed and estimates of metabolites determined by ELISA as described in the Methods. Values are in arbitrary units (ratio) ± SE. *a* = significantly different from LFD + supplement (*p* < 0.05) by ANOVA followed by Tukey's post-hoc test

In an attempt to correlate improvements in lipid profiles to changes in biomarkers of breast cancer risk, estradiol (E2), 2-hydroxyestrone (2-OHE1) and 16-α-hydroxyestrone (16-α-OHE1) were measured. Serum E2 results showed significant decreases in both the low-fat and habitual supplement phases as well as the washout phase, compared to baseline (Fig. 4A). There were no differences in NAF E2 at any intervention point or the washout (Fig. 4B). Despite similarities in the pattern of E2 levels in serum and NAF, when serum and NAF samples from the same participants were directly compared there was no correlation between the serum value and the NAF value, regardless of the time point of study (Fig. 4C). The ratio of 2-OHE1 to 16-α-OHE1 was significantly lower in the low-fat supplement period compared to the washout and habitual supplement phases, however this was not significantly different from baseline (Fig. 5).

## Discussion

This dietary intervention study was designed to examine the impact of n-3 FA supplementation, in the context of a low-fat compared to a higher-fat habitual diet, on the FA profile in healthy pre-menopausal women. Our hypothesis was that a long chain n-3 FA supplement (rich in DHA and EPA) would have a larger effect on lipid profiles when provided in the background of a low-fat diet. Fatty acids were examined in both the standard marker, red blood cells, as well as in nipple aspirate fluid to provide a measure of the changes at the target tissue.

Study participants, as shown in Table 1, were representative of a healthy pre-menopausal population of women. However, this group was atypical in that a substantial proportion of them had a family history of breast cancer (63 %), which is much higher than has been reported in other studies (12.5 % [29]). This observation may have contributed to the high level of compliance observed in the current study despite the fact that it was a lengthy protocol with minimal financial compensation. Thus, while a family history was likely a motivating factor for participation and compliance, it may limit the generalizability of the results.

Compliance with the low-fat dietary phase was very important in this study. Therefore, continuous 7-day food records were monitored to evaluate compliance. Since participants did not know in advance which records would be collected for detailed analysis, this limits the likelihood that they changed their eating behaviour at specific time points other than those specified in the study design. In total, three 7-day food records were used to examine habitual diet and three for low-fat diet phases. Compared to other food information collection approaches, this is a reliable and valid approach [30]. The process of recording also served to remind the participants to control their fat intake effectively. Analysis of the food records indicated that the participants came very close to the goal of 20 % dietary fat (22 % achieved) during the low-fat intervention period, resulting in a net decrease of 37 % dietary fat from their habitual diets. On average, women were consuming 39 g of fat during the low-fat diet phase, which is similar to the typical Japanese diet we were aiming to mimic (31 g; [31]). In the exit survey, only 2 participants indicated that the low-fat diet was particularly difficult to adhere to. To the contrary, most of the participants showed good tolerance to the low-fat diet and expressed a preference to consume a lower-fat diet in the future, instead of their habitual diet. Though participants were compliant, it is unlikely that the levels of DHA and EPA provided by the supplement, could have been easily replaced with dietary sources. This would have required that fatty fish be consumed at 3–5 times per week, and a major change in the diet pattern. Thus we would suggest that supplements would be the best choice for achieving these kinds of changes in membrane lipids at target tissues.

Compliance with the n-3 FA supplementation was also excellent with over 90 % consumption of the supplements provided. Consultation with the participants and examination of their diaries indicated very few side effects associated with either the dietary changes or supplement use. We do not have data to indicate whether the women indeed followed either the low-fat diet or continued with fish oil supplement use after study cessation.

The present study showed that a low-fat diet supplemented with n-3 caused a significant decrease in body weight, BMI, percent body fat, waist circumference, hip circumference, waist to hip ratio. Interestingly, body weight and BMI increased following the habitual diet with supplementation. While statistically there were no differences in total energy between the low-fat and habitual diets, there is significant error in estimating daily energy intake, and small differences may have been obscured by the large SDs. Thus it is possible that estimating energy intake was a less sensitive indicator using the methods herein, than absolute change in body weight over the three-menstrual cycle period (approximately 3 months). It is also possible that there were changes in physical activity or other behaviours as a result of the additional attention being given to the participants during the low-fat diet phase, despite the fact that they were being counselled not to change any other of their habits. Alternatively, the change in macronutrient composition associated with the low-fat diet may have resulted in increased energy expenditure, leading to a weight loss in these participants as has been reported before [32].

Before participants entered the intervention phase of the study, the fatty acid profile of RBC membranes was analyzed and again after the low-fat diet in all participants and in a random smaller sample of participants after the habitual supplement phase. Because there were no differences in the fatty acid composition of RBCs in Group 1 versus Group 2 participants, we conclude that the three menstrual cycle washout was sufficient to normalize their fatty acid levels before the second intervention period. Given the very large changes in n-3 FA composition in all participants, this gave a secondary indication of compliance along with the capsule count. Baseline distribution of the main fatty acids in RBC membranes in our study, were similar to that reported in a German cross-section study, which examined the association between allergic sensitization with n-3 PUFAs in the diet and in RBC membrane composition [33]. The baseline distribution was also similar to another American study based on a healthy population in Arizona [34]. However, the present study differs from a Swedish cohort study [35] examining postmenopausal women. The latter showed higher DHA and EPA levels, lower AA and a higher n-3:n-6 ratio, than those observed in the present study. One possible reason for this discrepancy could be baseline differences in FA consumption, or differences in menopausal status. Alternatively, these two groups may have had other differences in eating habits or other lifestyle factors (i.e. exercise) that could affect FA metabolism.

In the present study, DHA levels in RBC membranes were elevated by 58 % and EPA levels by 230 % following n-3 supplementation in the background of the low-fat diet (39 g/d). A decrease in AA was also observed but to a lesser extent (7.5 %) than for the long-chain PUFAs. In a similar randomized trial conducted by Geppert et al. [36] examining DHA supplementation alone (0.94 g DHA/d for 8 weeks) in vegetarians, DHA total FAs were increased, whereas EPA levels rose only marginally with no change in AA levels. The larger increase in EPA levels in the present study suggests that the change in EPA composition results from direct replacement of some membrane AA from the supplemental EPA provided (800 mg) as opposed to retroconversion of DHA to EPA and subsequent incorporation. The small change in AA observed here (12.96 ± 1.28 vs. 11.98 ± 1.55) is consistent with the study by Yuen and coworkers (13.2 vs. 11.5; [34]) (31). Review of several human studies [37, 38] suggests that supplementation with various EPA and DHA preparations produces a rise in AA concentrations when the total dose of EPA and DHA is relatively small, and that the commonly reported fall in AA concentration occurs only when the cumulative amount of EPA and DHA administered is high. The longer duration of supplementation (3 months) and relatively high dose of n-3 FAs (1.2 g/d), likely produced the AA decrease shown in our study.

Although both n-3 and n-6 FA intake from food sources were decreased in the low-fat diet compared to the habitual diet with supplementation (1.06 ± 0.47 vs. 0.64 ± 0.33 in n-3 FAs; 7.03 ± 2.84 vs. 4.69 ± 2.38 in n-6 FAs), no significant difference in the ratio of n-3 to n-6 FAs (0.51 ± 0.02 vs. 0.46 ± 0.007) was observed between these to experimental arms. This suggests that vegetable and other oils were removed from the diet rather than being substituted with other versions. Hence, the significant increase in n3:n-6 detected in RBC membrane can be specifically attributed to the n-3 FA supplement. Additionally, there was a consistent increase in the proportion of DPA (22:5n–3) in RBC membranes during both supplement periods. This suggests a possible up-regulation of elongase in response to the n-3 rich fatty acid supplementation. Because EPA can be converted into DPA by elongase, increased level of EPA from supplementary source provided more substrate for this metabolism.

To our knowledge, we are the first to report the effects of n-3 supplementation on nipple aspirate fluid. Our data demonstrate that n-3 supplementation has a direct effect on NAF, with increases in n-3 s and improvements in the n3:n6 ratio similar to that seen in RBCs. This indicates that the supplement is reaching its target tissue and therefore could be modulating its beneficial effects at the tissue of interest. As seen with RBCs, the increase in EPA and total n-3 s was also significantly higher on the background of a LFD in NAF.

Interestingly, despite the fact that the n-3:n-6 FA ratios were not significantly different between the low-fat supplemented and habitual supplemented diet phases in either RBCs or NAF, there were significant differences in the pools of these FAs, with the low-fat diet showing enhanced incorporation of n-3 FAs compared to the habitual diet. This suggests that the total level of fat in the diet has some direct impact on fatty acid partitioning in addition to the recognized importance of fatty acid ratio. A similar study to detect the effect of a low-fat diet on FA composition in RBCs found that FA profile responded more to a low-fat diet than a habitual diet [39]. It was also reported that n-6 FAs in RBCs were increased significantly following a low-fat diet intervention compared with a habitual diet. However, with the n-3 FAs supplements, our study showed no difference in total n-6 FA percentages between the low-fat and habitual diets. This inconsistency suggests that n-3 FAs supplementation could modify the FA profiles to a greater extent in the background of a low-fat diet. A mechanism to explain this differential partitioning remains unknown.

Although the functional roles of different RBC phospholipids may not be well known, we do know that changes in FA composition affect cell function [40], and therefore could potentially impact breast cancer development. Thus it is reasonable to predict that the FA stores in breast tissue may be similarly altered which could be reflected in the changes in NAF FA composition we observed; consequently, the breast cancer risk may be reduced. Indeed, in a recent study in men undergoing prostatectomies, n3 supplementation was demonstrated to favourably affect n3, n6 and n3:n6 in the prostate tissue, mirroring changes in RBC membrane PL content [41].

In an attempt to demonstrate an improvement in markers of breast cancer risk following the various arms of this study, metabolites of estrogen were measured. A positive correlation between serum estrogens and breast cancer risk in post-menopausal women is fairly well established [42, 43], but this is not the case for pre-menopausal women. Some association has been reported in pre-menopausal breast cancer [44], including one prospective study which found a positive association [45] while in five others there was no significant association between circulating estrogen levels and breast cancer risk [42]. Additionally, the ratio of urinary 2-OHE1 to 16-α-OHE1 as a breast cancer risk marker has been suggested [46], however a recent review found only a weak positive association between this ratio and breast cancer risk in pre-menopausal but not post-menopausal populations. We observed a significant decrease in serum E2 from baseline during both supplementation periods as well as during the washout. While 3 months is usually considered sufficient to "washout" red blood cell fatty acids, it is not clear whether this is sufficient time to re-establish baseline hormone levels, that could potentially take much longer. There were no significant changes in NAF E2 concentrations despite changes in FA composition in this tissue. There was also no correlation between serum and NAF E2 concentrations suggesting that changes could occur in one compartment without occurring in the other. The suggestion has been made that the NAF (or other tissue of breast origin) is a better place to measure biomarkers of breast cancer risk, particularly estrogen metabolites [42] and it is quite possible that urinary or circulating concentrations of estrogen metabolites may not be good surrogate biomarkers. However, our very low success rate with recovering NAF, despite having well-trained and dedicated personnel to collect it, and very good participant compliance with the procedure, suggests that substitution of NAF for serum for routine screening, is not practical. Furthermore, until the at-risk metabolic profile (possibly achieved through more recent proteomic methods [47–49]) can be determined, there will be little opportunity to determine whether diet or other lifestyle factors have the ability to affect risk at the tissue level.

## Conclusions

We have shown that the influence of long-chain n-3 fatty acids on tissue FA composition is affected by the total fat level in the diet; low-fat diets are associated with better incorporation of DHA and EPA than traditional Western diets. Importantly, we have demonstrated that these improvements are measurable in a surrogate of the target tissue, nipple aspirate fluid. Although others [50, 51] have suggested that there are no positive associations between fat intake or specific fatty acid intake and breast cancer, is important to note that there were very few participants consuming a very low-fat diet in one [50], and a small range in intake of fatty acids in the other [51]. We would argue that the potential benefits of long-chain n-3 fatty acids can only be realized in the background of a low-fat diet and at levels of supplementation that most closely represent the traditional Japanese diet, where better correlations between diet and breast cancer risk has been identified [8, 36]. We did see a significant decrease in circulating estrogen levels with supplementation and this could be associated with a decreased cancer risk in these pre-menopausal women, consistent with some previous studies. There was, however, no decrease in NAF hormone levels and thus it is possible that any benefit of the n-3 fatty acids is occurring through a mechanism unrelated to NAF E2. Omega-3 fatty acids can act on a number of metabolic pathways and newer metabolomic and proteomic approaches may be able to identify more appropriate candidate biomarkers.

## Abbreviations

16-α-OHE1: 16-α-hydroxyestrone
2-OHE1: 2-hydroxyestrone
AA: Arachidonic acid
BMI: Body mass index
DHA: Docosahexaenoic acid
E2: Estradiol
EPA: Eicosapentaenoic acid
FA: Fatty acid
LFD: Low fat diet
MC: Menstrual cycle
MUFA: Monounsaturated fatty acid
NAF: Nipple aspirate fluid
PL: Phospholipid
PUFA: Polyunsaturated fatty acid
RBC: Red blood cell
